# PHYH c.678+5G>T Leads to In-Frame Exon Skipping and Is Associated With Attenuated Refsum Disease

**DOI:** 10.1167/iovs.65.2.38

**Published:** 2024-02-27

**Authors:** Malena Daich Varela, Elena Schiff, Samantha Malka, Genevieve Wright, Omar A. Mahroo, Andrew R. Webster, Michel Michaelides, Gavin Arno

**Affiliations:** 1 Moorfields Eye Hospital, London, United Kingdom; 2 UCL Institute of Ophthalmology, University College London, London, United Kingdom; 3 Great Ormond Street Hospital for Children, London, United Kingdom

**Keywords:** Refsum, PHYH, genetics, genomics, retina

## Abstract

**Purpose:**

To investigate the molecular effect of the variant *PHYH*:c.678+5G>T. This variant has conflicting interpretations in the ClinVar database and a maximum allele frequency of 0.0045 in the South Asian population in gnomAD.

**Methods:**

We recruited patients from Moorfields Eye Hospital (London, UK) and Buenos Aires, Argentina, who were diagnosed with retinitis pigmentosa and found to have biallelic variants in *PHYH*, with at least one being c.678+5G>T. Total RNA was purified from PaxGene RNA-stabilized whole-blood samples, followed by reverse transcription to cDNA, PCR amplification of the canonical *PHYH* transcript, Oxford Nanopore Technologies library preparation, and single-molecule amplicon sequencing.

**Results:**

Four patients provided a blood sample. One patient had isolated retinitis pigmentosa and three had mild extraocular findings. Blood phytanic acid levels were normal in two patients, mildly elevated in one, and markedly high in the fourth. Retinal evaluation showed an intact ellipsoid zone as well as preserved autofluorescence in the macular region in three of the four patients. In all patients, we observed in-frame skipping of exons 5 and 6 in 31.1% to 88.4% of the amplicons and a smaller proportion (0% to 11.3% of amplicons) skipping exon 6 only.

**Conclusions:**

We demonstrate a significant effect of *PHYH*:c.678+5G>T on splicing of the canonical transcript. The in-frame nature of this may be in keeping with a mild presentation and higher prevalence in the general population. These data support the classification of the variant as pathogenic, and patients harboring a biallelic genotype should undergo phytanic acid testing.

Inherited retinal diseases (IRDs) are a heterogeneous group of genetic disorders that lead to subnormal vision, primarily due to abnormalities in photoreceptors and retinal pigment epithelium cells.^1^ IRDs may develop as an isolated ocular condition or as part of a syndrome, accompanied by a range of extraocular manifestations.^2^ Visual symptoms of syndromic IRDs may arise before systemic manifestations (e.g., *CLN3*–Batten disease),^3^ at the same time (e.g., *SCA7*–spinocerebellar ataxia),^4^ or after (e.g., Usher syndrome type 1).^5^ In recent years, wider accessibility of genetic testing has led to the diagnosis of attenuated or milder forms of syndromic IRDs, often before systemic signs or symptoms are identified.^6^ Moreover, studies have identified variants in genes usually associated with syndromic disease causing isolated retinal disease (e.g., *HGSNAT*, *MFSD8*, *CLN3, IFT140*).^7^^–^^10^ Retinitis pigmentosa (RP) is the most common IRD, affecting approximately 1 in 3500 to 4000 individuals.^11^^,^^12^

Refsum disease (phytanic acid storage disorder) is a rare syndrome initially characterized by four clinical signs: RP, peripheral neuropathy, cerebellar ataxia, and increased proteins in the cerebrospinal fluid.^13^^,^^14^ Less frequent manifestations include anosmia, miotic and poorly reactive pupils, hearing loss, ichthyosis, skeletal abnormalities in feet and hands, and cardiac arrhythmia.^15^ Onset of symptoms is often in the second to third decades of life, with initial presentation of night blindness, unsteady gait, poor sense of smell, and peripheral weakness. Individuals with Refsum disease were found to have increased blood and tissue levels of phytanic (3,7,11,15-tetramethylhexadecanoic) acid and undetectable levels of the peroxisomal enzyme, phytanoyl-CoA hydroxylase (PhyH).^16^^,^^17^ *PHYH* (MIM *602026) was subsequently identified as the causative gene, encoding PhyH, the enzyme catalyzing the first step in phytanic acid α-oxidation.^18^ More recently, damaging variants in *PEX7* (peroxin 7, PhyH protein transporter into peroxisomes) have also been associated with a minority of cases of Refsum disease.^19^^,^^20^

To date, 37 pathogenic and likely pathogenic variants have been reported in *PHYH* in the Human Gene Mutation Database,^21^ accessed in October 2023. No genotype–phenotype correlations have been identified, including no association between levels of phytanic acid and visual acuity.^15^ Jansen et al.^22^ first reported disease associated variants in *PHYH*. They described three splice-site variants with skipping of exon 6 (182 nucleotides deletion) leading to a reading frame-shift and a premature stop codon: c.678+2T>G, c.679-1G>T, and c.678+5G>T. The authors suggested that exon 6 is a structurally important element and that variants located here cause loss of enzymatic activity.^22^

In recent years, the variant *PHYH*:c.678+5G>T has received conflicting interpretations (ClinVar ID: 198192).^23^ It has an overall frequency of nearly 1 in 1000 in the general population, higher in South Asians and Ashkenazi Jewish (>4 in 1000), with five homozygotes (gnomAD v4.0.0). However, it has also been found in *trans* with other variants in patients with RP*.*^22^^,^^24^ Here, we used long-read cDNA amplicon sequencing to determine the splicing effect of the variant *PHYH*:c.678+5G>T.

## Methods

Patients were identified from the Inherited Eye Disease clinics at Moorfields Eye Hospital (MEH, London, UK). Individuals diagnosed with an IRD by ophthalmic genetic specialists were offered clinical genetic testing, performed using panel-based targeted next-generation sequencing, exome sequencing, or genome sequencing. Five patients from five unrelated families who were found to have biallelic variants in *PHYH*, with at least one of them being c.678+5G>T, were recruited. An additional patient (6) was recruited from Buenos Aires, Argentina, following review of his genetic testing results by the authors, identifying biallelic variants in *PHYH*, one of them being c.678+5G>T.^25^ Informed consent was obtained from all patients. Ethical approval was provided by the local ethics committee and the study honored the tenets of the Declaration of Helsinki. Where appropriate and available, blood samples were taken from parents or siblings to confirm segregation of proposed variants. All individuals and their genetic testing results were discussed in multidisciplinary team meetings, other candidate genes and variants were ruled out, and *PHYH*:c.678+5G>T was selected for further investigation.^26^

For the targeted transcript sequencing experiment, blood samples were collected from patients and (when available) family members after obtaining informed consent. Total RNA was purified from PAXgene stabilized whole blood (Qiagen, Hilden, Germany) followed by reverse transcription to cDNA using random hexamers and SuperScript IV reverse transcriptase (Invitrogen, Carlsbad, CA, USA). PCR was performed using primers designed to capture the canonical transcript (NM_006214.4) from exon 1 to –3′UTR (forward CAGATTGTTCTGGGCCACCT; reverse ACACCGTTCCTATGCCCTTG). RNA from healthy controls was also included. After PCR and gel electrophoresis, 5 µL PCR product was purified using 9 µL Ampure XP (Beckman Coulter Inc., Brea, CA, USA) magnetic beads and resuspended in 10 µL Qiagen elution buffer. Qubit High Sensitivity dsDNA quantification was performed and the molar concentration of the sample was estimated. Up to 30 fmol of amplicon was used for end preparation (Ultra II end-prep kit; NEB, Ipswitch, MA, USA) and native barcoding (Oxford Nanopore Technologies; ONT: EXP-NBD104, Oxfordshire, UK). Barcoded samples were pooled (up to 12 samples per run) and library preparation performed using the ONT ligation sequencing kit SQK-LSK110 Flongle protocol. Libraries were sequenced using the Flongle flowcell and MinION sequencer and runs were typically 12 to 24 hours.

Basecalling was performed using the fast basecalling or high-accuracy basecalling mode in Minknow or Guppy v.5.0.16 after the run was completed. Adaptor sequences were removed using Porechop v.0.2.4 (https://github.com/rrwick/Porechop), and reads were filtered using NanoFilt v.2.8.0 for reads 800 to 1800 bp with a quality score ≥Q10.^27^ Resulting FastQ data were aligned to the human reference genome (build GRCh38) using minimap2 v.2.22 (https://github.com/lh3/minimap2).^28^ BAM files were generated using SAMtools v.1.9 (http://www.htslib.org/).^29^ JVarKit biostar214299 was used to split the BAM files by single-nucleotide variant (SNV) genotype (http://github.com/lindenb/jvarkit). The Integrative Genome Viewer v.2.7.2 and v.2.14.1 were used to visualize aligned long-read data sets and generate sashimi plots for splice junction analysis.^30^

Relevant patient data were retrieved from the electronic health care record and imaging software systems. Patients were categorized using the World Health Organization visual impairment criteria, which define no or mild visual impairment as best-corrected visual acuity (BCVA) ≤0.48 (6/18, 20/60), moderate impairment as BCVA >0.48 and ≤1.0 (6/60, 20/200), severe as BCVA >1.0 and ≤1.3 (3/60, 20/400), and blindness as BCVA >1.3 (https://icd.who.int/en). Only BCVA was taken into consideration to classify patients. Clinical assessments consisted of dilated fundus examination, spectral-domain optical coherence tomography (Heidelberg Spectralis; Heidelberg Engineering, Inc., Heidelberg, Germany), fundus autofluorescence (Heidelberg Spectralis; Heidelberg Engineering and Optos PLC, Dunfermline, UK), and ultrawide field fundus color photography (Optos PLC). Very long-chain fatty acid blood test for peroxisomal function, specifically phytanic acid level, was undertaken in different available clinical laboratories.

## Results

In total, nine unrelated patients were identified in the MEH database with biallelic likely disease-causing variants in *PHYH* and an associated isolated or syndromic IRD. Of these, five had c.678+5G>T in the homozygous or heterozygous state, and their clinical phenotype is detailed below. An additional patient (6) with biallelic variants in *PHYH* and c.678+5G>T in the heterozygous state was recruited from Argentina, and his details are also described. Four of these, patients 1 to 4, provided a blood sample for transcript analysis (Table). In addition, one carrier of *PHYH*:c.678+5G>T (unaffected son of patient 4) and three controls were included in the RNA experiments. Additional variants of uncertain significance found in the patients are detailed in Supplementary Table S1.

**Table.** Characteristics of the Patients in the Study

	*PHYH*													
ID	Variant 1	Variant 2	Age First Visit, Y	Age Onset, Y	Symptom Onset	VA First Visit	Syndromic?	Phytanate Levels, µmol/L	VA Last	Age Last, y	FU Time	Provided Blood for Long-Read RNA Sequencing?	Transcripts Skipping Exon 6, %	Transcripts Skipping Both Exons, %
1	c.678+5G>T	c.823C>T, p.Arg275Trp	27	12	Night blindness	6/24 and 6/12	Anosmia lifelong	10.62; normal up to 10 (normalized after diet)	6/7.5 and 6/9 (post phaco)	36	9	Yes	0	31.1
2	c.678+5G>T	c.103delT, p.Ser35ProfsTer19	44	10	Night blindness	6/5 OU	None	Normal (11.12; normal up to 15)	6/7.5 and 6/12	64	20	Yes	6.9	53.2
3	c.678+5G>T	c.467C>A, p.Thr156Lys	63	20	Night blindness	6/18 and HM	Anosmia since 20s, peripheral neuropathy	259.2; normal up to 15	6/24 and PL	65	2	Yes	2.2	39.2
4	c.678+5G>T	c.678+5G>T	50	NA	Asymptomatic	6/6 OU	Anosmia since childhood	Normal	6/9 and 6/18	75	25	Yes	11.3	88.4
5	c.678+5G>T	c.678+5G>T	39	29	Decreased night and peripheral vision	6/7.5 OU	None	NA	NA	NA	0	No	NA	NA
6	c.678+5G>T	c.823C>T, p.Arg275Trp	79	25	Decreased night and peripheral vision	6/48 and 6/19	Hiposmia, hearing loss since age 55 y, and peripheral neuropathy	Normal	NA	NA	0	No	NA	NA
Carrier	c.678+5G>T	NA	—	—	—	—	—	—	—	—	—	Yes	0	55.1
Control	—	—	—	—	—	—	—	—	—	—	—	Yes	0	4.5
Control	—	—	—	—	—	—	—	—	—	—	—	Yes	0	2
Control	—	—	—	—	—	—	—	—	—	—	—	Yes	0	2

All patients had one allele c.678+5G>T, and the second allele is specified in the table. 6: Argentinian individual. FU, follow-up; HM, hand movements; Hom, homozygous; NA, not available or applicable; OU, both eyes; phaco, phacoemulsification (cataract surgery); PL, perception of light; VA, visual acuity.

### Clinical Characteristics

**Figure 1.**
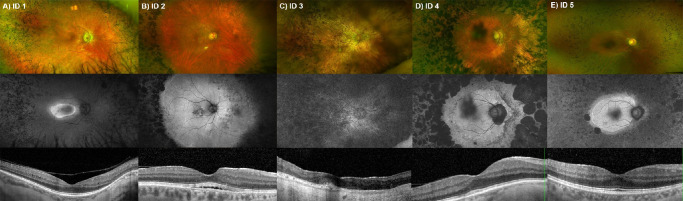
Retinal images of patients with RP associated with *PHYH*:c.678+5G>T. (**A**) Patient 1 at 34 years old. Ultra-widefield imaging (Optos PLC) is positive for a hyperautofluorescent ring at the macular region and fine pigmented bone spicule–like deposits in the periphery. Macular spectral-domain optical coherence tomography (OCT) shows an overall preserved macular architecture, with present macular EZ line. (**B**) Patient 2 at 62 years old. Optos imaging shows clear sparing of the retinal posterior pole, with deep pigmented deposits in the periphery. Macular OCT scan demonstrates subretinal fluid and preserved EZ line. (**C**) Patient 3 at 63 years old. Retina-wide atrophy, with scattered pigmented deposits, loss of autofluorescence, and a small remnant of EZ line. (**D**) Patient 4 at 71 years old. Retinal imaging shows patches of atrophy mainly in the midperiphery, along with deep pigmented deposits, both sparing the posterior pole. OCT scan shows largely preserved macular EZ line. (**E**) Patient 5 at 39 years of age. Optos imaging is positive for mild pigment deposits in the midperiphery and vessel thinning. Autofluorescence highlights a preserved posterior pole, with hypoautofluorescence in the periphery. Macular OCT shows continuous EZ.

Patient 1 (GC20955) is a white male who was first seen at 27 years of age. He reported having night blindness from approximately 12 years of age and being diagnosed with RP in his teenage years. There was no family history of eye disease. BCVA at age 27 years was logMAR 0.6 right eye (OD) and 0.3 left eye (OS). He had bilateral cataracts, and his fundus features were of typical RP, with bone spicule–like (BSL) deposits, vessel thinning, and a hyperautofluorescent ring in the posterior pole (Fig. 1). He had a preserved island of ellipsoid zone (EZ) at the macula and visually nonsignificant edema in both eyes. He was reportedly fit and well. Following his molecular diagnosis, he mentioned having lifelong reduced sense of smell. Phytanic acid levels were mildly above threshold (10.62 µmol/L, 0.62 µmol/L above the upper limit of the acceptable range), and Refsum disease was also confirmed through a skin biopsy. At age 36, his vision had improved to logMAR 0.1 OD and 0.2 OS after cataract surgery (phacoemulsification). There were no significant changes in his retinal examination.

Patient 2 (GC15871) is a white female first seen at 44 years of age. She reported night blindness from age 10 years and was reportedly fit and well, with no affected family members. A full-field electroretinogram (ERG) performed at age 44 years showed mild amplitude reduction and normal implicit time of rod responses, as well as borderline cone responses. Pattern ERG (PERG) suggested macular dysfunction. Her vision was logMAR –0.1 OD and OS, and she had bilateral clear visual axis and BSL pigment deposits in the retinal periphery. She had continuous macular EZ and developed subretinal fluid at age 60 years, without any evidence of a choroidal neovascular membrane. Follow-up ERG at age 62 years revealed overall stable full-field and worsening of the PERG, suggesting decreased macular function in both eyes. Genetic testing was positive for *PHYH*:c.678+5G>T and c.103delT. She denied longstanding poor smell or neurologic problems, but she was found to adopt a flat-footed gait for stability. She also had poor iris dilation. On her last visit, at age 64 years, she had logMAR 0.1 OD and 0.3 OS, and she had passed the visual field test for driving eligibility and was driving. There were no significant changes in her retinal examination, with remaining bilateral subfoveal fluid and largely preserved posterior pole outer retina. Phytanic acid testing was normal (11.12 µmol/L, normal up to 15 µmol/L) at 64 years of age.

Patient 3 (GC28415) is a white female who was first seen at age 63 years. She remembered having night blindness from around the beginning of the third decade of life and was diagnosed with RP at age 31 years. There were no other affected family members. Her medical history was positive for progressive peripheral neuropathy, cardiac arrythmia, loss of smell, and asthma. On examination, she had logMAR 0.5 OD and hand movements OS visual acuity. She had poor dilation and was pseudophakic in both eyes, and there was a significant retinopathy with small islands of retained retina in both eyes, with widespread atrophy involving the macula. Refsum disease was suspected since her initial visit given the syndromic characteristics. Phytanic acid in blood was markedly elevated (259.2 µmol/L, nearly 20-fold the normal upper limit of 15 µmol/L). Genetic testing confirmed two variants in *PHYH*, c.678+5G>T and c.467C>A, and segregation confirmed that these were in *trans*. On her last visit, at age 65 years, her vision and ocular examination were largely unchanged.

Patient 4 (GC20417) is a white male who was first seen at age 50 years. He was found to have an unusual retina in a routine examination and did not report any visual difficulties. His medical history was positive for ischemic heart disease and hyperlipidemia, and there were no known affected relatives. On examination, he had logMAR 0 OD and OS, minor lens opacities, and BSL deposits in the retinal periphery, with a largely spared posterior pole. At age 70 years, *PHYH*:c.678+5G>T was found in homozygosity, and he mentioned having a longstanding poor sense of smell. Phytanic acid levels were found to be within normal limits. On his last visit, at age 75 years, his visual acuity was 0.2 OD and 0.5 OS, he had mild cataracts, and there were no significant changes in his retinal examination.

Patient 5 (GC21338) is an Asian male who was first seen at 39 years of age. He was found to have retinal changes in a routine eye examination in his late 20s and described possibly having decreased night and peripheral vision compared to his peers. He had type 2 diabetes and hypertension. He had a distant cousin who also reportedly had RP. On examination, he had logMAR 0.1 OD and OS, dilated pupil diameters of approximately 9 mm, mild bilateral cataracts, midperipheral BSL changes, and continuous macular EZ. ERG and PERG revealed mild generalized retinal dysfunction, with macular involvement. Genetic testing revealed *PHYH*:c.678+5G>T in homozygosity, and phytanic acid testing is to be undertaken.

Patient 6 is a white male who described having noticed decreased night and peripheral vision since around 25 years old. He did not have any affected family members, and his medical history consisted of adult-onset hearing loss (requiring hearing aids) and peripheral neuropathy, causing distal decreased sensitivity and ataxia. Although he was not aware of issues with his sense of smell, his wife mentioned he is often not able to notice smells around the house. His BCVA at age 79 years was logMAR 0.9 OD and 0.5 OS, and his retinal examination showed a widespread retinopathy involving the macula. After genetic testing revealed *PHYH*:c.823C>T, phytanic acid testing was undertaken and was found to be within normal limits (undetectable levels). Analysis of the patient's BAM file by the authors revealed the second variant, c.678+5G>T.

### Long-Read RNA Sequencing and Splicing Analysis

The c.678+5G>T variant allele led to skipping of exons 5 and 6 in 31.1% to 55.1% of the reads generated by RT-PCR in heterozygotes and approximately 88.4% skipping in the homozygous patient 4 (Table). Low-level skipping of only exon 6 was also observed, ranging from 0% of reads in individual 1 to 11.3% of reads in patient 4. Control individuals (*n* = 3) showed 2% to 4.5% of the reads lacking exons 5 and 6, corresponding to an in-frame alternatively spliced transcript (variant 5, NM_001323083.1), with no reads skipping exon 6 alone.

**Figure 2.**
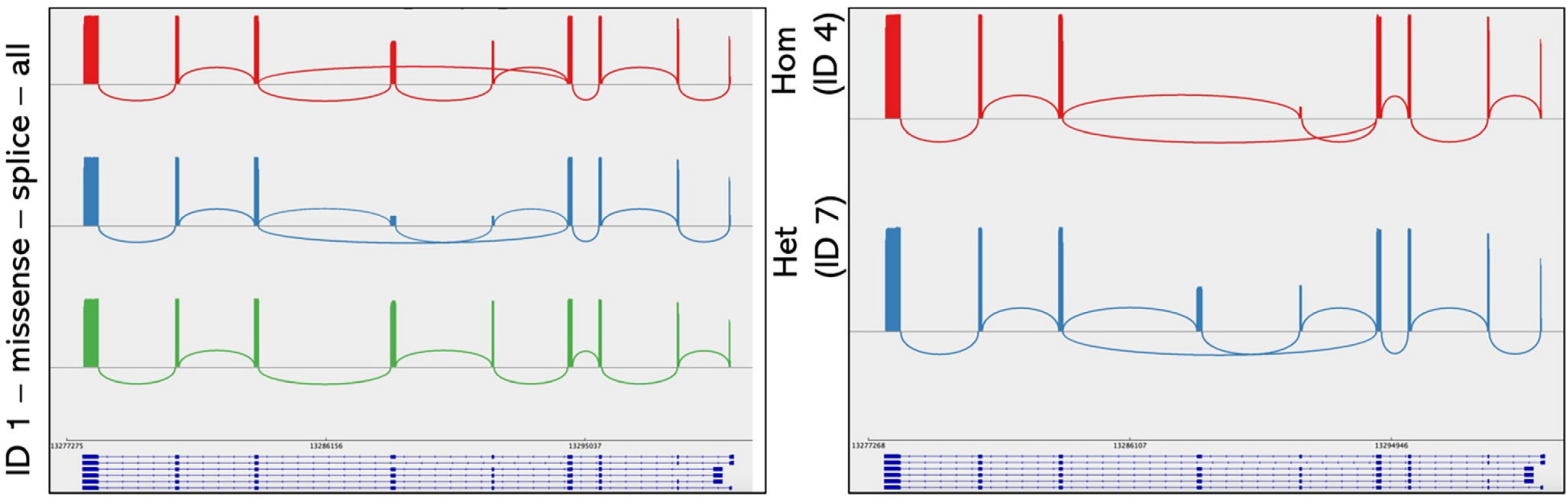
Sashimi plot of patients 1 (*left*) and 4 and 7 (*right*) showing splice junctions of *PHYH* transcript. *Left*: red represents total reads, showing 31.1% of the reads skipping exons 5 and 6. Blue corresponds to the c.678+5G>T allele, with 15% of reads including exons 5 and 6, and green is the *trans* allele with normal splicing. *Right*: red represents the data for individual 4 (homozygous), with only 11.6% of the transcripts including exon 5 and 0.3% exon 6. Blue corresponds to 7, the heterozygous carrier offspring of 4, with 55.1% reads skipping exons 5 and 6.

As the splice variant is noncoding, the read data were phased using single-nucleotide variants on the *trans* allele where possible (patients 1, c.678+5G>T; c.823C>T and 3, c.467C>A; c.678+5G>T). For patient 1, of the 412 reads surviving filtering, 262 (63.6%) were the c.823T allele and 150 (36.4%) were the c.823C allele harboring the c.678+5G>T variant (^1^Fig. 2). This skew in reads generated from each allele may represent a difference in transcription level or may have arisen from PCR allele preference. Analysis of these phasing data showed that 128 of 150 (85.3%) c.823C allele reads skipped exons 5 and 6, with 22 reads (14.7%) including exons 5 and 6, possibly indicating incomplete loss of the wild-type splicing on the c.678+5G>T allele. No reads from the *trans* allele showed detectable skipping.

For patient 3 with a missense variant located in exon 5 and no other coding SNV, similar phasing was not possible. Analysis of reads showed that 938 of 2395 reads (39.2%) skipped exons 5 and 6, and 52 (2.2%) skipped exon 6 only. Of the reads including exons 5 and 6, 132 of 1405 (9.4% or 5.5%) arose from the c.467C allele harboring the c.678+5G>T variant and thus it can be estimated that 90.3% of this allele underwent exon skipping.

Examination of the read data from individual 2 (c.103delT on the *trans* allele) showed 692 of 1301 reads (53.2%) skipped exons 5 and 6, with 90 reads (6.9%) skipping exon 6 only. The read data for individual 4 (homozygous) showed skipping of exons 5 and 6 on 6511 of 7365 reads (88.4%, Fig. 2) and skipping of exon 6 in a further 11.3% of the reads, suggesting the primary effect of c.678+5G>T in this individual is the in-frame deletion of exons 5 and 6 with a near-complete loss of exon 6. His heterozygous carrier offspring was also tested (Fig. 2), showing skipping of exons 5 and 6 on 286 of 519 (55.1%) reads. Additional phasing for these individuals was not possible due to the absence of coding SNVs.

The alternate transcript annotated as NM_001323083.1 excludes exons 5 and 6, which leads to the in-frame deletion of 264 bp from the transcript and 88 residues from the polypeptide sequence if translated. In contrast, deletion of exon 6 alone (seen in very low transcript levels from these studies) would lead to deletion of 182 bp and out-of-frame splicing between exons 5 and 7, leading to a reading frame shift (p.Lys167GlyfsTer3) and early termination as described by Jansen et al.^22^

## Discussion

This study demonstrates the splice effect of *PHYH*:c.678+5G>T, highlighting its importance in *PHYH*-associated disease. This rare intronic variant currently has six interpretations in ClinVar (ID 198192): four uncertain, one likely pathogenic, and one (the most recent) likely benign. The variant is mentioned in five studies in the literature, reported in at least five patients.^22^^,^^24^^,^^31^^–^^33^ Yet, its interpretation in the clinical setting is uncertain, leading to this variant often not being reported by clinical laboratories.

The pathogenicity of variants with high prevalence is often questioned. Two examples of common IRD variants causing milder disease when in *trans* with certain changes are *ABCA4* p.Asn1868Ile and *CNGB3* p.Arg403Gln.^34^^–^^37^ *ABCA4*:p.Asn1868Ile has a worldwide prevalence of >5%, highest in Europeans, with over 2750 homozygotes (https://gnomad.broadinstitute.org/). It is estimated that it accounts for around 10% of the cases and ∼80% of late-onset presentations of Stargardt.^38^ *CNGB3*:p.Arg403Gln has been identified in >2% of South Asian chromosomes with 63 homozygotes (https://gnomad.broadinstitute.org/). It has been associated with macular dystrophy, cone dystrophy, and achromatopsia in certain genotypes, and it is thought to modify the CNG channel conduction properties.^39^

*PHYH*:c.678+5G>T is rarer but commonly found in patients with *PHYH*-associated disease, even in the homozygous state. This study demonstrates that *PHYH*:c.678+5G>T primarily causes in-frame skipping of exons 5 and 6, in contrast to the previously described frameshifting effect.^22^ The overall splicing effect was variable, ranging from approximately 85.3% in patient 1 to 99.7% in patient 4 transcripts altered, indicating possible incomplete abolition of the splice donor site (SpliceAI prediction: 0.35 -moderate donor loss-).^40^ Our analysis was able to characterize transcripts found in whole blood. Retina-specific splicing may not be well represented here, but if the canonical transcript is the predominant one in the neural retina, we expect the same effect. The mild ocular phenotype, limited systemic features, and high prevalence in the general population may suggest that this is a milder/hypomorphic allele. Furthermore, this report constitutes an additional case in which long-read sequencing has provided key information to characterize a complex variant. Long-read or third-generation sequencing methods such as Pacific Biosciences (PacBio) and ONT have shown promising results in detecting structural variants and disease-causing variants in repeat expansions,^41^^,^^42^ as well as characterizing RNA splicing isoforms.^43^^,^^44^ The increasing accuracy and decreasing cost are making these techniques a viable option to possibly overcome some of the difficulties faced with short-read sequencing.^45^ In comparison to quantitative PCR or droplet digital PCR, our method of RT-PCR amplicon long-read sequencing enables the full characterization of the splicing landscape in a single reaction but is currently limited to being qualitative or semiquantitative.

Patients with RP often describe the onset of symptoms (night blindness, constricted field) in the first or second decade of life,^46^ and it can take up to 30 years until the diagnosis is made.^15^ By this time, the degree of retinal degeneration is usually severe, with most patients having a 20-degree field.^47^ It is remarkable that the visual acuity and appearance of most of the patients analyzed in this report were on the milder side of the spectrum, with two of them having a spared posterior pole at a relatively advanced age (ID 2 and 4), even when in *trans* with a null allele (ID 2).

The ophthalmologic signs and symptoms in Refsum disease tend to manifest early on, with patients often complaining of night blindness before additional symptoms arise (mean age of onset 16 years).^15^ There are limited data about the ophthalmologic phenotype of patients with Refsum disease, but it appears that they can have peripheral pigment that progresses toward the macula, variable BCVA, rapidly constricting fields (with 75% of eyes having less than 20 degrees remaining at the time of diagnosis), and severely diminished ERG responses.^15^^,^^48^ Even though the ocular phenotype was variable among the patients described herein, it appears that in both patients 2 and 5 who had ERG testing (in the seventh and fourth decades of life), the retinal function was not as affected as in the literature.^15^^,^^48^ Taking into account the preserved EZ in patients 1, 2, 4, and 5, the estimated visual field would correspond to more than 30 degrees in all of them.^49^ The mean age of onset of the cohort was also later than the previously described (19.2 years).^15^ It is unclear why patients 3 and 6 had a more severe phenotype than the rest of the patients, but it appears that most patients described herein (1, 2, 4, and 5) had a milder presentation, consistent with the hypomorphic allele.

Early detection and management of a retinal dystrophy are helpful in mitigating its impact on affected individuals’ quality of life. Moreover, unlike most other IRDs, for Refsum disease, there are therapeutic interventions that can be applied, often involving dietary restrictions by reducing the intake of phytanic acid. In addition, treatments targeting the metabolic pathways involved in phytanic acid metabolism are currently under investigation.^50^^,^^51^ It is interesting that four of the patients had normal to near-normal levels of phytanic acid, one with a null allele in *trans*. This may point to a hypomorphic or possibly tissue/isoform-specific effect of this intronic variant, with the retina more sensitive to it. The retina has high metabolism and is particularly sensitive to oxidative damage,^52^^,^^53^ and phytanic acid has been shown to have a cytotoxic effect with increased reactive oxygen species.^54^ In patients with suspected Refsum disease but normal phytanic acid levels in blood, skin, nerve, or liver biopsy can be useful to confirm the diagnosis.^55^ In conclusion, we present data that demonstrate the pathogenicity of *PHYH*:c.678+5G>T and its effect on the transcript, in keeping with the milder presentation and higher prevalence in the general population. These data will contribute toward improved molecular diagnoses, particularly relevant in one of the few IRDs with management options.

## Supplementary Material

Supplement 1
