# Supplementary material for: PHYH c.678+5G>T Leads to In-Frame Exon Skipping and Is Associated With Attenuated Refsum Disease
Source: Invest Ophthalmol Vis Sci. 2024 Feb 27;65(2):38. doi: 10.1167/iovs.65.2.38 (PMC10910431; doi:10.1167/iovs.65.2.38)
Supplement: Supplement 1 [file iovs-65-2-38_s001.docx]

| **ID** | **Additional variants identified** |
| --- | --- |
| 1 | *ERCC6* c.3061A>G p.Ile1021Val het  *CDHR1* c.526-7C>G het  *CTSD* c.127G>C p.Val43Leu het  *ARAP1* c.2120C>T p.Ala707Val het  *OCA2* c.1465A>G p.Asn489Asp het  *RLBP1* c.796-6C>T het  *MYO7B* c.3371C>A p.Thr1124Lys het  *GPR98* c.17204+4A>G het  *MEOX2* c.205C>A p.His69Asn het  *KIAA1549* c.5228C>T p.Thr1743Met het  *MT-ATP6* c.313G>A p.Ala105Thr |
| 2 | *CACNA2D4* c.2095C>T p.(Leu699Phe) het  *CC2D2A* c.1392C>A p.(Gly464Gly) het  *FSCN2* c.522C>A p.(Leu174Leu) het  *GRM6* c.1875C>T p.(Tyr625Tyr) het  MKKS c.59A>G p.(GIu20Gly) het  *NMNATI* c.126A>C p.(Thr42Thr) het  *PANK2* c.666G>A p.(Leu222Leu) het  *RPE65* c.168A>G p.(Pro56Pro) het  *VPS13B* c.7753G>A p.(Glu2585Lys) het |
| 3 | NA |
| 4 | *ERCC6* c.1337G>A p.Gly446Asp het  *OAT* c.-30+8C>T het  *MYO7A* c.2587T>C p.Tyr863His het  *OCA2* c.1849A>T p.Ile617Leu het  *SLC24A1* c.931G>C p.Val311Leu het  *MPRIP* c.3395G>C p.Glu1132Asp het  *HADHA* c.620T>C p.Leu207Ser het  *ALMS1* c.7630G>T p.Ala2544Ser het  *LRP2* c.3355C>A p.His1119Asn het  *IDH3B* c.117+6C>A het  *PDE6B* c.496G>A p.Glu166Lys het  *BBS12* c.1859A>G p.Gln620Arg het  *DNAH5* c.8757G>C p.Glu2919Asp het  *CTNNA1* c.618G>C p.Gln206His het  *KIAA1549* c.3052A>T p.Ile1018Leu het  *HGSNAT* c.427G>A p.Gly143Arg het |
| 5 | NA |
| 6 | *DSCAML1* c.4300A>G p.(Ile1434Val) het  *NRL* c.375C>G p.(His125Gln) het |
